# Confidence-based classification of clinically significant prostate cancer on biparametric MRI using Monte Carlo dropout and Grad-CAM visualization

**DOI:** 10.3389/fonc.2026.1819986

**Published:** 2026-10-07

**Authors:** Cheng Hao, Yuze Meng, Yong Peng, Xia Du

**Affiliations:** Department of Radiology, the Affiliated Hospital of Guizhou Medical University, Guiyang, China

**Keywords:** biparametric MRI, confidence analysis, gradient-weighted class activation mapping, Monte Carlo dropout, prostate cancer

## Abstract

**Objective:**

Accurate early diagnosis of Clinically Significant prostate cancer (csPCa) is crucial for improving patient survival rates. This study aimed to develop and validate an interpretable, confidence-based deep learning model for csPCa classification using biparametric MRI (bpMRI).

**Methods:**

A retrospective analysis was conducted on bpMRI data from 410 patients. A classification model was constructed using the visual geometry group 16-layer (VGG-16) network, incorporating gradient-weighted class activation mapping (Grad-CAM) for visual interpretability, Monte Carlo Dropout (MC-Dropout) for uncertainty estimation, and a rejection mechanism. The model’s performance was evaluated using receiver operating characteristic (ROC) curves and area under the ROC curve (AUC).

**Results:**

Across all predictions, the confidence-based model achieved an overall accuracy of 79.3% before rejection. At a confidence threshold of 0.8, 80.8% of predictions were retained, among which the selective accuracy was 94.6%, with 99 false-positive and 2 false-negative predictions. Besides, the model’s accuracy outperformed that of a senior radiologist (94.6% vs 90.8%). Qualitative Grad-CAM visualization demonstrated that the classifier frequently assigned high activation to MRI regions considered suspicious for csPCa; however, quantitative spatial agreement with lesion annotations was not assessed.

**Conclusion:**

This confidence-based model identifies csPCa lesions and provides reliable confidence scores without pixel-level annotations. By rejecting uncertain predictions, it enhances the transparency and safety of clinical decision-making.

## 1 Introduction

Prostate cancer (PCa) is the second most prevalent malignancy in men worldwide, with an incidence second only to lung cancer (1). In China, the incidence of PCa has risen steadily in recent years, primarily due to an aging population (2). As early-stage clinically significant PCa (csPCa) is frequently asymptomatic, timely screening and precise diagnosis are essential for improving patient survival outcomes.

Magnetic resonance imaging (MRI) has become one of the most important tools for csPCa diagnosis. The internationally recognized Prostate Imaging-Reporting and Data System (PI-RADS) recommends multiparametric MRI (mpMRI) as the standard imaging method. The mpMRI typically consists of three sequences: T2-weighted imaging (T2WI), diffusion-weighted imaging (DWI), and dynamic contrast-enhanced imaging (DCE). However, DCE requires intravenous administration of contrast agents, which increases examination cost and procedural complexity, and carries potential risks of nephrotoxicity and allergic reactions. These limitations restrict its widespread use in large-scale population screening and routine follow-up.

Therefore, biparametric MRI (bpMRI), which excludes DCE and relies solely on T2WI and DWI, has gained increasing attention and adoption in clinical studies for csPCa evaluation (3). As these two modalities performed without contrast injections, bpMRI substantially expands the applicability of MRI-based csPCa diagnosis. Previous studies have demonstrated that bpMRI achieves diagnostic performance comparable to that of mpMRI, while offering favorable practicality and reliability (4). However, interpretation of bpMRI images remains highly dependent on the radiologist’s experience and expertise and is susceptible to inter-reader variability, fatigue, and differences in training background (5, 6).

To address these challenges, deep learning-based computer-aided diagnosis (CAD) systems have achieved remarkable progress in csPCa image analysis. With the rapid development of convolutional neural networks (CNNs), deep learning method has become one of the mainstream techniques in csPCa imaging analysis. These methods can automatically extract informative features from MRI, significantly improving diagnostic efficiency and accuracy, and have the potential to reduce variability arising from manual reading (7, 8). However, despite the greatly improved classification accuracy of current deep learning models, challenges of model interpretability and prediction reliability remain inadequately addressed (9, 10). In clinical practice, these models usually cannot provide a case-wise confidence level for each prediction, rendering it difficult for physicians to rely on the model to support precise decision-making.

Monte Carlo Dropout (MC-Dropout) is a deep learning technique that enables uncertainty estimation. By applying random dropout multiple times at test time, it can provide reliable confidence scores for model predictions, thereby enhancing transparency and clinical interpretability of deep learning outputs (11). This approach facilitates the identification of regions with high predictive uncertainty, enhances reliability assessment, and assists clinicians in more effectively understanding the model’s decision-making process.

Therefore, in this study, we propose and validate a confidence-based model for classifying benign versus malignant prostate lesions using MC-Dropout. Our aim is to accurately discriminate benign from malignant prostate lesions using bpMRI while providing a confidence score for each prediction, thereby improving the accuracy and safety of clinical decision-making.

## 2 Materials and methods

### 2.1 Patients

This retrospective study included patients who underwent prostate MRI and had histopathological confirmation at the Department of Radiology, Affiliated Hospital of Guizhou Medical University from February 2023 to August 2025. A total of 410 patients were enrolled, comprising 267 patients with PCa and 143 patients without PCa, yielding 2303 images for analysis.

The inclusion criteria for PCa patients were as follows: (1) no prior prostate biopsy or prostate surgery before MRI; (2) prostate biopsy performed within 2 weeks after MRI; (3) no history of radiotherapy, chemotherapy, or hormonal therapy; (4) complete and diagnostically adequate imaging data (T2WI and DWI) as well as PI-RADS and Gleason scores. The inclusion criteria for non-PCa patients included either histopathological confirmation from biopsy or a minimum of 6-month clinical and imaging follow-up without evidence of malignancy. This study was approved by the institutional ethics committee (approval no. 2305104). Considering the retrospective design of this study, the requirement for written informed consent was waived.

### 2.2 Imaging protocol

All MRI examinations were performed on a 3.0 T Discovery 750W scanner (GE Healthcare, USA) using a 16-channel phased-array body coil. Patients were scanned in the supine position, head-first, with the imaging volume covering the entire prostate.

For model construction and classification, only axial T2WI and axial DWI were used, with the following parameters: Axial T2WI: repetition time/echo time (TR/TE) = 3716/125 ms; slice thickness = 4 mm; interslice gap = 0 mm; spatial resolution = 0.7 × 0.9 × 4 mm³. Axial DWI: TR/TE = 5000/82 ms; slice thickness = 4 mm; interslice gap = 0 mm; spatial resolution = 2.7 × 2.4 × 4 mm³; *b* = 0, 800, and 1500 s/mm². Apparent diffusion coefficient (ADC) maps were automatically generated by the scanner using the *b* = 0 and *b* = 800 s/mm².

### 2.3 Model construction

#### 2.3.1 Data preprocessing

To minimize the influence of surrounding pelvic structures on deep learning performance, we first used the automatic prostate segmentation method proposed by Zhu et al. (12) to extract the prostate gland. The segmentation method was not retrained or independently quantitatively validated in the present cohort. Voxels outside the segmented prostate were set to zero, and a rectangular bounding box was cropped surrounding the prostate based on the centroid of the segmentation mask.

The cropped images were then linearly normalized using adaptive histogram equalization. The processed T2WI, high-*b*-value DWI, and ADC images were subsequently combined to form a 3-channel image stack (T2WI + DWI + ADC). To augment the training data, random affine transformations were applied to these 3-channel stacks, effectively tripling the number of training samples.

Histopathology from transrectal ultrasound-guided biopsy served as the reference standard. The csPCa was defined as Gleason score ≥3 + 4/ISUP Grade Group ≥2, whereas benign findings and Gleason score 3 + 3/ISUP Grade Group 1 were categorized as non-csPCa. For patients with csPCa, two experienced prostate radiologists, with 7 and 9 years of experience in prostate MRI diagnosis, independently and blindly reviewed the axial images to determine the presence of MRI-visible csPCa on each slice. Disagreements were resolved by consensus. Accordingly, the individual axial three-channel bpMRI slice (T2WI, DWI, and ADC) served as the unit of classification and subsequent performance evaluation; no pixel-level lesion annotations were used for model training.

For comparison with human interpretation, a senior prostate radiologist with 11 years of dedicated prostate MRI experience independently reviewed the bpMRI examinations. The reader was blinded to histopathological results, model outputs, and the original radiology reports. No clinical information was provided. The reader assigned PI-RADS scores according to PI-RADS v2.1. For patient-level evaluation, the highest PI-RADS score within each examination was used, with PI-RADS ≥4 considered positive for csPCa and PI-RADS ≤3 considered negative.

#### 2.3.2 Network architecture

We adopted the classical VGG-16 network as the backbone architecture (13). VGG-16 consists of 13 convolutional layers, 3 fully connected layers, and several dropout layers. The model input was a 3-channel prostate image, resized by bilinear interpolation to 224 × 224 pixels, and the output was a binary classification probability generated by a sigmoid activation function.

To incorporate prior visual knowledge, the convolutional layers were initialized with ImageNet pretrained weights, whereas the fully connected layers were trained from scratch using the collected dataset.

The network was optimized using Adam with an initial learning rate of 1x10^-4^, batch size of 16, and a maximum of 100 epochs. Binary cross-entropy was used as the loss function. No specific class-balancing strategy was applied.

To provide a stronger architectural baseline, a ResNet-50 classifier was implemented using the same three-channel T2WI, high-b-value DWI, and ADC inputs. ResNet-50 was trained and evaluated using the same preprocessing procedures, patient-level cross-validation splits, and classification labels as VGG-16, thereby allowing a controlled comparison between backbone architectures.

#### 2.3.3 Model interpretability and confidence analysis with Grad-CAM and MC-dropout

To visualize the decision process of the classification network, the gradient-weighted class activation mapping (Grad-CAM) was integrated into the trained VGG-16 model. Grad-CAM calculates the gradient of the class score with respect to the feature map activations and performs global average pooling over width and height to obtain a weight for each feature map (14). A weighted sum of feature maps yielded a localization map, highlighting the key regions for classification.

During inference, dropout remained active in two dropout layers, with a dropout probability of 0.5. Fifty stochastic forward passes were performed for each input image.

To further enable confidence analysis, MC-Dropout was applied on top of the Grad-CAM-enhanced model to quantify predictive uncertainty and construct an explainable csPCa classification system with confidence scores. During testing, two dropout layers remained active, with a dropout probability of 0.5. Fifty stochastic forward passes were performed for each input image. The resulting 50 classification outputs and 50 corresponding activation maps were then aggregated.

For image-level classification, the mean of the *n* predictions was taken as the final probability. The predictive variance *D* across the *n* predictions was used to define a confidence score *C*. Considering the most extreme binary case, in which half of the predictions are 0 and half are 1, yielding a maximum variance of 0.25, we defined a normalized confidence score *C* as:


$$C=1-(\frac{D}{0.25})$$

where *D* denotes the predictive variance. Thus, *C* ∈ [0, 1], with higher values indicating higher confidence.

For activation maps, the mean and confidence score were calculated for each pixel across the *n* Grad-CAM maps, yielding a confidence-weighted activation map. According to a predefined confidence threshold, predictions with confidence below the threshold were labeled as “uncertain, “ whereas those meeting or exceeding the threshold were accepted as valid benign or malignant classifications. A schematic diagram of the overall model workflow is illustrated in **Figure 1**.

**Figure 1:**
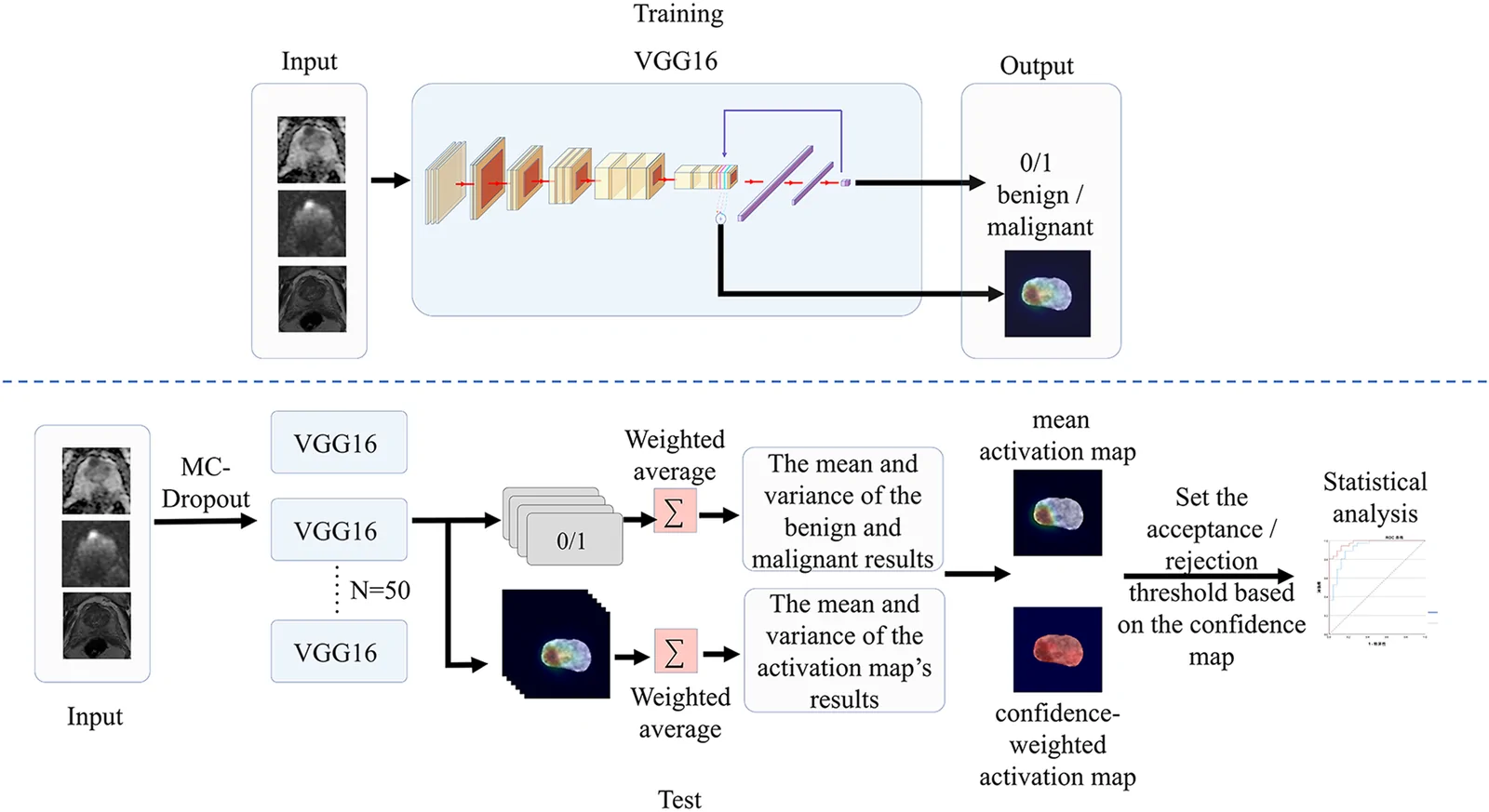
Flowchart of the confidence-based and interpretable PCa classification model.

### 2.4 Statistical analysis

All statistical analyses were performed using MedCalc. Five-fold cross-validation was performed strictly at the patient level. All patients were divided into five mutually exclusive folds, and all axial slices belonging to the same patient were assigned to the same fold. In each iteration, four folds were used for model training and the remaining fold was used for held-out validation. Therefore, no patient or slice appeared simultaneously in both the training and validation sets within an individual iteration. Across the five iterations, each patient served as a held-out validation case exactly once. Final performance was calculated by pooling the out-of-fold slice-level predictions from the five held-out folds. No separate independent test cohort was used.

For each model, the overall accuracy as well as the number of false-positive (FP) and false-negative (FN) predictions were calculated. Chi-square tests were used to assess differences in classification performance between models.

Receiver operating characteristic (ROC) curves were plotted for both models, and the area under the ROC curve (AUC) was computed. The DeLong test was applied to compare AUCs and to determine whether the confidence-based model with a rejection option greatly outperformed the original VGG-16 model. A two-sided *P* value < 0.05 was considered statistically significant.

All reported diagnostic performance metrics were calculated at the individual-slice level; no patient-level aggregation of slice-level predictions was performed.

## 3 Results

### 3.1 Qualitative results

**Figure 2** shows a representative example of a high-confidence prediction in a typical patient with csPCa (Gleason score 4 + 4). **Figures 2A–C** displays a single slice of T2WI, DWI, and the corresponding ADC map, respectively. **Figure 2D** demonstrates the Grad-CAM activation map overlaid on the image. The red high-activation region corresponds to the true tumor location, indicating that the proposed model can correctly learn lesion features using only image-level labels (benign vs malignant). **Figure 2E** presents the confidence-weighted activation map, in which most regions exhibit confidence values close to 1, suggesting the model is highly confident in both the localization and classification of this lesion.

**Figure 2:**
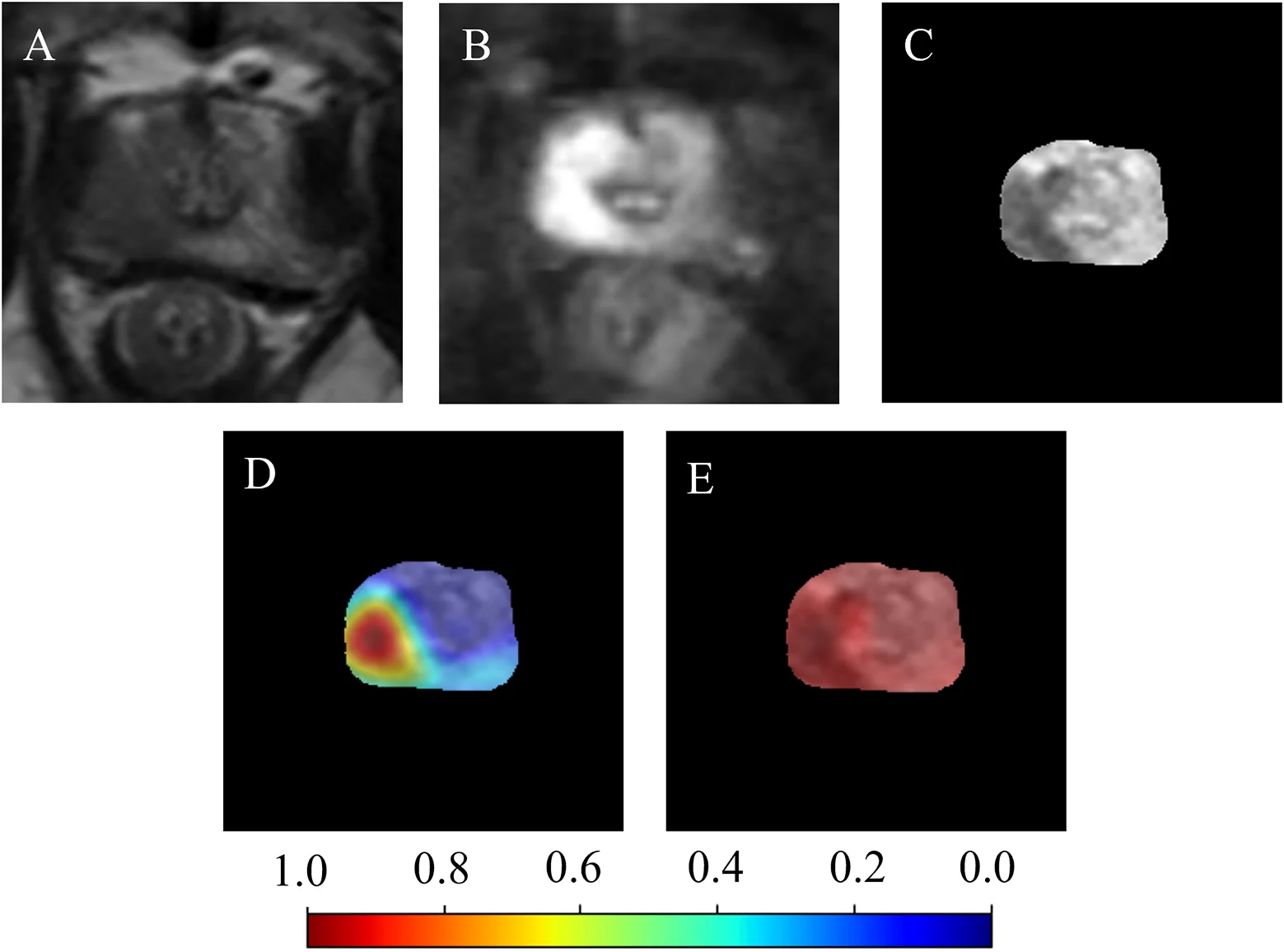
An example of visualization for a case with high model confidence. **(A–C)** T2-weighted image, diffusion-weighted image and corresponding ADC map of a patient with PCa. **(D)** Model’s interpretable activation map. **(E)** Confidence-weighted activation map.

In contrast, **Figure 3** illustrates a case with low model confidence. **Figures 3A–C** displays a single slice of T2WI, DWI, and the corresponding ADC map, respectively. Although **Figure 3D** shows certain high-activation regions, these regions exhibit low confidence in the corresponding map (**Figure 3E**), indicating that the model’s uncertainty regarding whether these areas are truly cancerous. Consequently, such predictions were rejected by the model. Notably, this case represents a benign lesion that was misclassified by original VGG-16 model. The rejection of low-confidence predictions effectively prevents such errors from being accepted as final outputs.

**Figure 3:**
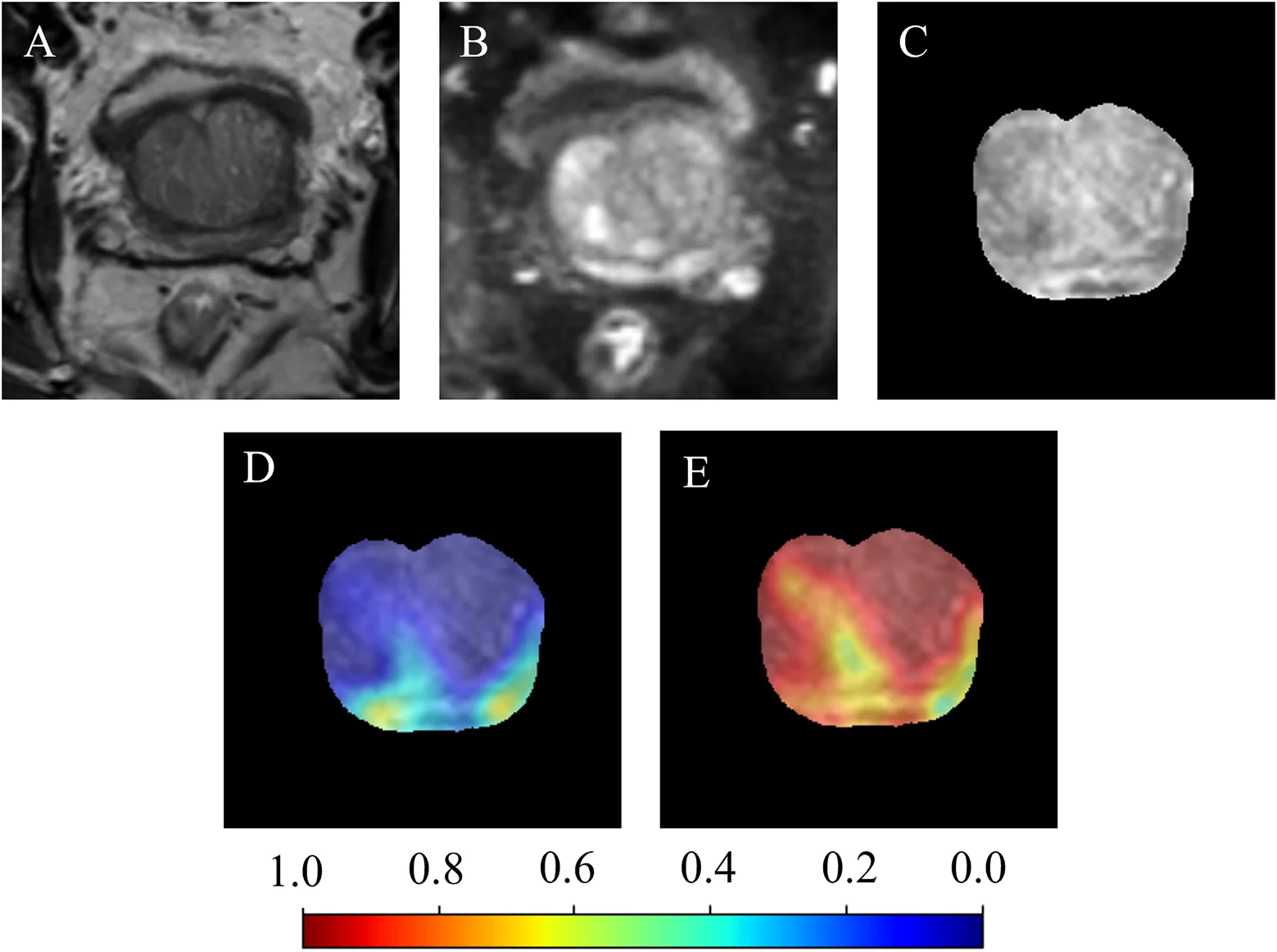
An example of visualization for a case with low model confidence. **(A–C)** T2-weighted image, diffusion-weighted image and corresponding ADC map of a patient. **(D)** Model’s interpretable activation map. **(E)** Confidence-weighted activation map.

### 3.2 Quantitative results

Out-of-fold predictions were obtained for all 2, 303 images from 410 patients through five-fold patient-level cross-validation. As an additional architectural baseline, ResNet-50 achieved an AUC of 0.90, compared with 0.87 for the original VGG-16 classifier and 0.93 for the proposed confidence-based framework. ResNet-50 achieved an accuracy of 82.9%. The ROC curves of the three models are shown in **Figure 4**.

**Figure 4:**
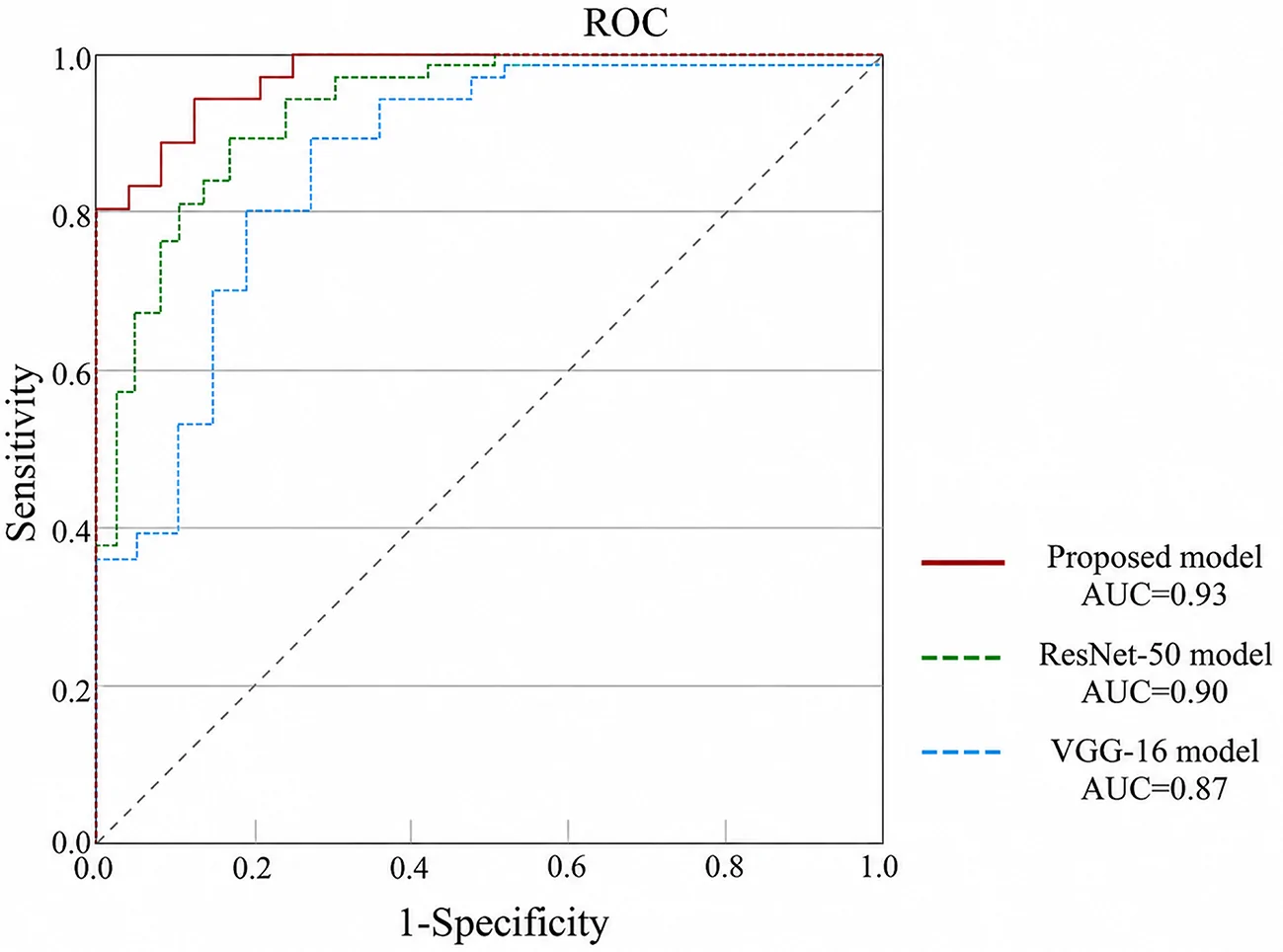
Comparison of ROC curves among the proposed model, ResNet-50 and the original VGG-16 model.

For each image, the model produced a classification result accompanied by an MC-Dropout derived confidence score. Based on repeated experiments, a confidence threshold of 0.8 was determined to provide the optimal balance of performance. Predictions with confidence < 0.8 were labeled as uncertain and rejected, whereas those with confidence ≥ 0.8 were accepted.

Across all 2, 303 out-of-fold image predictions, the confidence-based model correctly classified 1, 825 images, with 350 false-positive and 128 false-negative predictions, corresponding to an overall accuracy of 79.3% before application of the rejection mechanism. At the confidence threshold of 0.8, 1, 861 images (80.8%) were accepted as high-confidence predictions, whereas 442 images (19.2%) were rejected as low-confidence predictions. **Table 1** summarizes the accuracy, False Positive (FP) rate, and False Negative (FN) rate, for the senior radiologist, the original VGG-16 model, and the confidence-based model across both accepted and rejected subsets.

**Table 1:** Comparison of classification results among senior radiologist, the original VGG-16 model and the confidence-based model.

Classification results	Senior radiologist	VGG-16 model	Confidence-based model	
			Rejected results	Accepted results
FP (%)	126 (5.5)	352 (15.3)	251 (56.7)	99 (5.3)
FN (%)	86 (3.8)	125 (5.4)	126 (28.5)	2 (0.1)
Correct classification (%)	2 091 (90.8)	1 826 (79.3)	65 (14.8)	1 760 (94.6)

VGG-16, visual geometry group 16-layer; FP, False Positive; FN, False Negative.

Among the rejected cases, FP accounted for the majority (56.7%). Certain predictions were rejected despite being correct, due to low confidence scores. Among the 1, 861 accepted high-confidence predictions, 1, 760 were correctly classified, corresponding to a selective accuracy of 94.6%. The accepted subset contained 99 false-positive and 2 false-negative predictions. These values represent performance conditional on acceptance at the confidence threshold and should therefore be interpreted together with the corresponding coverage of 80.8%, rather than as overall performance across all images. For high-confidence predictions, the overall accuracy of the model surpassed that of the senior radiologist (94.6% vs 90.8%).

Chi-square analysis revealed a statistically significant difference in classification performance between the confidence-based model and the original VGG-16 model (*χ*² = 199.88, *P* < 0.001), demonstrating that integrating MC-Dropout-based confidence thresholds markedly improved accuracy and stability.

The ROC analysis further demonstrated that the AUC of the proposed model was higher than that of the original VGG-16 model and ResNet-50 (*P* < 0.05; DeLong test).

## 4 Discussion

Early and accurate diagnosis of csPCa is critical for improving patient survival. The bpMRI has emerged as a key modality for csPCa screening and diagnosis in recent years because of its avoidance of contrast injections, ease of operation, and shorter acquisition time. However, efficiently and automatically extracting lesion information from bpMRI while achieving high sensitivity and specificity remains a major clinical challenge.

With the rapid development of deep learning, CAD systems have achieved remarkable advances in medical image analysis (15). By leveraging data-driven feature learning from MRI, deep learning methods shift diagnostic assessment from subjective visual interpretation toward a more objective and reproducible process, thereby improving diagnostic performance and minimizing reader-dependent variability (16). A recent multicenter study involving 10, 207 patients reported that a deep learning model, compared with 62 experienced radiologists, reduced the FP rate by approximately 50% at matched sensitivity and improved the detection rate of csPCa. The model’s overall AUC was significantly higher than that of human readers (0.91 vs 0.86) (17). A 2024 systematic review further reported that, when radiologists applied PI-RADS v2.1 with thresholds of ≥ 3 or ≥ 4 for csPCa diagnosis, the specificity ranged from 0.60 to 0.82, with FP rates still as high as 18%–40% (18). These findings highlight the diagnostic advantages of artificial intelligence models and suggest that introducing effective CAD systems may substantially reduce unnecessary prostate biopsies and improve overall diagnostic accuracy.

However, most current deep learning-based CAD systems rely on high-quality pixel-level annotations. For example, Abraham et al. (19) used a pretrained VGG-16 network to grade PCa on bpMRI, achieving satisfactory performance. Song et al. (7) proposed a patch-based CNN model to classify cancerous versus noncancerous tissue patches in prostate images. Schelb et al. (8) adapted U-Net, originally designed for segmentation, to perform pixel-wise classification in PCa bpMRI. Although these methods can capture fine-grained lesion information, generating pixel-level annotation is labor-intensive, time-consuming, and prone to inter-observer variability, which may lead to high FP rates and limit clinical scalability.

In contrast, labeling at the patient or slice level is more cost-effective, but models trained in this manner typically produce only binary outputs (cancer versus non-cancer) without explaining the underlying decision. Such models cannot reveal whether predictions are driven by true lesion regions, nor can they indicate the reliability of each prediction. To improve transparency, interpretability methods such as Grad-CAM have been introduced in medical imaging (20–23). Grad-CAM visualizes network activation regions and helps clinicians understand the model’s focus. Although these methods provide image-level explanations, they still generate a single deterministic prediction for each case, even for challenging and out-of-distribution samples. The absence of explicit confidence information limits their practical utility in clinical decision-making.

To address these gaps, we developed a bpMRI-based csPCa classification model that integrates MC-Dropout and Grad-CAM. Our model simultaneously improved classification performance, quantified prediction confidence, and offered visual explanations of decision regions. Through rigorous 5-fold cross-validation, we demonstrated the effectiveness of MC-Dropout: at a confidence threshold of 0.8, the performance of accepted predictions was markedly improved compared with that of the original VGG-16 model. Chi-square tests confirmed a significant difference in accuracy (*P* < 0.001), whereas ROC analysis with the DeLong test demonstrated a higher AUC for the confidence-based model (*P* < 0.05).

To provide a stronger architectural comparison, we additionally evaluated ResNet-50 under the same experimental setting. Its AUC of 0.90 was higher than that of the original VGG-16 model (0.87), but remained below that of the proposed confidence-based framework (0.93). These findings suggest that the observed performance was not solely attributable to comparison with a relatively simple VGG-16 baseline. Nevertheless, the present study was not designed as a comprehensive benchmark of contemporary prostate MRI architectures. Rather, our primary objective was to investigate whether predictive uncertainty could identify potentially unreliable model outputs. Future studies should evaluate the proposed uncertainty-aware framework using transformer- and foundation-model-based architectures in larger multicenter cohorts.

We further evaluated the behavior of the confidence-based rejection mechanism. At the confidence threshold of 0.8, approximately 80.8% of predictions were retained as high-confidence predictions, among which the classification accuracy was 94.6%. Importantly, this higher accuracy should not be interpreted as an unconditional improvement in model performance. Rather, it reflects a selective prediction strategy in which lower-confidence cases are withheld at the expense of reduced diagnostic coverage. Thus, the selective accuracy of 94.6% should be interpreted together with the corresponding coverage of 80.8%. The rejected subset contained a substantially higher proportion of misclassified predictions, suggesting that MC-Dropout-derived confidence may help identify potentially unreliable model outputs. A complete characterization of selective classification would require evaluation across multiple operating thresholds using risk-coverage analysis, which was beyond the prespecified scope of the current proof-of-concept study.

Grad-CAM was used exclusively as a *post-hoc* visualization tool and did not influence the classification probability or the confidence-based acceptance/rejection decision. Therefore, changes in selective classification performance were attributable to MC-Dropout-based uncertainty estimation and the associated rejection mechanism rather than Grad-CAM. In representative cases, Grad-CAM highlighted image regions visually associated with areas considered suspicious for csPCa on bpMRI. However, because standardized lesion-level annotations were unavailable, quantitative spatial agreement was not assessed. Accordingly, the activation maps should be interpreted as qualitative visual explanations rather than validated lesion-localization maps.

Analysis of low-confidence misclassified cases revealed that benign inflammatory or hemorrhagic regions can exhibit low signal intensity on ADC maps similar to that of malignant lesions, potentially confounding both the model and human readers. These findings underscore the need for clinicians to pay special attention to these atypical cases and interpret model outputs in conjunction with clinical context.

The limitations of this study must be acknowledged. First, the dataset was derived from a single center and a single 3.0T scanner, which may limit the generalizability of the findings. In future work, multicenter validation is planned, including data from different institutions using various scanners and involving more diverse patient cohorts. Second, diagnostic performance was evaluated at the axial-slice level rather than at the lesion or patient level. Consequently, the reported performance should not be directly interpreted as patient-level diagnostic performance. Because no patient-level aggregation strategy was predefined, we did not retrospectively optimize an aggregation rule in the present study. Future studies should prospectively define lesion- and patient-level aggregation strategies and evaluate corresponding diagnostic performance. Third, small lesions (usually < 5 mm) may pose challenges for the model, as subtle lesion-related signal changes can be partially obscured by complex glandular anatomy or volume effects. Future work will explore the integration of attention mechanisms to enhance the model’s sensitivity to subtle lesion regions and improve spatial feature discrimination. Fourth, although a previously validated prostate segmentation algorithm was used for preprocessing, its segmentation accuracy was not independently quantified in the current dataset. Segmentation errors could potentially influence downstream classification and should be systematically assessed in future validation studies. Last, the use of 2D architecture represents another limitation. The retrospective DWI data had anisotropic spatial resolution (2.7 × 2.4 × 4 mm³), and the cohort contained a relatively limited number of independent patients despite a larger number of individual slices. We therefore selected a 2D transfer-learning approach for this proof-of-concept study. Future larger multicenter datasets should evaluate 2.5D and fully 3D architectures to determine whether volumetric contextual information further improves diagnostic performance and uncertainty estimation.

## 5 Conclusion

In summary, this study presents a confidence-based bpMRI classification model for csPCa that combines MC-Dropout-based confidence analysis with Grad-CAM visualization. The proposed model substantially improves classification accuracy and reliability, effectively reduces the risk of clinical misdiagnosis, and provides transparent decision support for clinicians. Moreover, it eliminates the need for exhaustive pixel-level annotations, reducing labeling cost and enhancing scalability. This approach demonstrates promising clinical application and translational potential and may open new avenues for precise diagnosis and treatment decision-making in csPCa.

## Data Availability

The original contributions presented in the study are included in the article/supplementary material. Further inquiries can be directed to the corresponding author.
